# Role of Pre-therapeutic ^18^F-FDG PET/CT in Guiding the Treatment Strategy and Predicting Prognosis in Patients with Esophageal Carcinoma

**DOI:** 10.7508/aojnmb.2016.02.001

**Published:** 2016

**Authors:** Teik Hin Tan, Ching Yeen Boey, Boon Nang Lee

**Affiliations:** Department of Nuclear Medicine, National Cancer Institute, Putrajaya, Malaysia

**Keywords:** Esophageal carcinoma, FDG, PET/CT, Prognosis

## Abstract

**Objective(s):**

The present study aimed to evaluate the role of pre-therapeutic ^18^fluorine-fluorodeoxyglucose positron emission tomography-computed tomography (^18^F-FDG PET-CT) and maximum standardized uptake value (SUV_max_) in guiding the treatment strategy and predicting the prognosis of esophageal carcinoma, using the survival data of the patients.

**Methods:**

The present retrospective, cohort study was performed on 40 consecutive patients with esophageal carcinoma (confirmed by endoscopic biopsy), who underwent pre-operative ^18^F-FDG PET-CT staging between January 2009 and June 2014. All the patients underwent contrast-enhanced CT and non-contrasted ^18^F-FDG PET-CT evaluations. The patients were followed-up over 12 months to assess the changes in therapeutic strategies. Survival analysis was done considering the primary tumor SUV_max_, using the Kaplan–Meier product-limit method.

**Results:**

In a total of 40 patients, ^18^F-FDG PET-CT scan led to changes in disease stage in 26 (65.0%) cases, with upstaging and downstaging reported in 10 (25.0%) and 16 (40.0%) patients, respectively. The management strategy changed from palliative to curative in 10 out of 24 patients and from curative to palliative in 7 out of 16 cases. Based on the ^18^F-FDG PET-CT scan alone, the median survival of patients in the palliative group was 4.0 (95% CI 3.0-5.0) months, whereas the median survival in the curative group has not been reached, based on the 12-month follow-up. Selection of treatment strategy on the basis of ^18^F-FDG PET/CT alone was significantly associated with the survival outcomes at nine months (P=0.03) and marginally significant at 12 months (P=0.03). On the basis of SUV_max_, the relation between survival and SUV_max_ was not statistically significant.

**Conclusion:**

^18^F-FDG PET/CT scan had a significant impact on stage stratification and subsequently, selection of a stage-specific treatment approach and the overall survival outcome in patients with esophageal carcinoma. However, pre-treatment SUV_max_ failed to stablish its usefulness in the assessment of patient prognosis and survival outcome.

## Introduction

The incidence of esophageal carcinoma in Malaysia is 1.5 per 100,000 populations (1). Esophageal carcinoma as a relatively rare disease is ranked 17^th^ and 22^nd^ most common cancer in Malaysia in males and females, respectively. Despite significant advances in the diagnosis and treatment of this disease, the overall five-year survival remains relatively poor (2). Therefore, accurate pre-therapeutic staging of esophageal carcinoma, which subsequently guides the stage-adapted treatment approach, is critical in optimizing the survival outcomes (3).

The location and depth of tumor involvement, together with the presence of nodal and systemic metastases, are important parameters in guiding treatment approaches such as radical curative surgery, definitive chemoradiotherapy, and palliative therapy (3). In order to fully assess the disease extent, a multimodality approach, comprised of endoscopic ultrasound (EUS), computed tomography (CT), and ^18^fluorine-fluorodeoxyglucose positron emission tomography-CT (^18^F-FDG PET/CT), is usually adopted.

For the initial tumor (T) staging, EUS has been shown to be the optimal modality in assessing the depth of transmural infiltration (4). Furthermore, this modality facilitates ultrasound-guided biopsy which is associated with a higher histopathological yield. For the evaluation of tumor extension, contrasted CT presents a well-demarcation in depicting local invasion to the adjacent structures and provides some information on peritumoral lymph node involvement (5).

For the evaluation of remote nodal and systemic metastases, which majorly dictate the therapeutic options, ^18^F-FDG PET/CT scan is particularly useful (6, 7). Furthermore, utility of ^18^F-FDG PET/CT scan has led to changes in esophageal cancer staging (3-5) and has been found to significantly affect patient management (6, 10).

Several studies have shown the prognostic value of standardized uptake value (SUV) in the overall survival of patients with esophageal carcinoma (11). However, this finding has not been replicated in other studies, and the significant impact of SUV_max_ alone on the overall patient survival has not been documented (12, 13). Therefore, the purpose of this study was to evaluate the role of pre-therapeutic ^18^F-FDG PET/CT and SUV_max_ in predicting the treatment strategy and prognosis of patients with esophageal carcinoma, using the survival data of the patients.

## Methods

The present retrospective, cohort study was performed on 40 consecutive patients with esophageal carcinoma (confirmed by endoscopic biopsy), who underwent pre-operative ^18^F-FDG PET/CT staging between January 2009 and June 2014. The histological findings, tumor location, prior contrasted CT scan findings, ^18^F-FDG PET/CT results, SUV_max_ of the primary lesion, post-PET/CT treatment, patient follow-up, and survival status of the patients were retrieved, using the hospital database and contacting the patients or their relatives.

The study protocol was approved by the local ethics committee, and informed consents were obtained from the patients through conversations with the patients and their relatives.

### ^18^F-FDG PET/CT imaging protocol

The patients were required to fast for at least four hours prior to examination. Upon admission, the patients’ body weight was measured and blood glucose level was recorded. Then, 6 MBq/kg of ^18^F-FDG (range: 285-460 MBq) was intravenously injected, and PET/CT imaging was performed on the dedicated GE^®^ Discovery ST Scanner, equipped with PET and eight-slice CT units. Image acquisition was performed with a whole-body field of view (vertex to mid-thigh), 45-60 min after the injection.

CT transmission images for attenuation correction were captured with exposure factors of 120 kVp, 80 mA, and 0.8 s for all examinations; no intravenous CT contrast was administered. Emission PET images were obtained in a two-dimensional mode at the rate of four min per bed position with a three-slice overlap between consecutive bed positions.

Transaxial PET data were reconstructed using filtered back-projection. The CT data for PET were reconstructed to axial slices with a thickness of 3.3 mm. The images were reviewed on GE Advantage Workstation (version 4.2) by two experienced nuclear medicine physicians. Positive uptakes on PET images were based on non-physiological uptakes greater than liver or SUV_max_>2.5, corrected for body weight.

### Determination of disease stage

All patients had undergone contrasted CT evaluation prior to ^18^F-FDG PET/CT scan. The median interval between CT and PET/CT scan was one month. Due to suboptimal spatial resolution on PET with noncontrast CT, no attempt was made to define local invasion or peritumoral lymphadenopathy during PET/CT interpretation. Therefore, on the basis of ^18^F-FDG PET/CT alone, information on T stage and N1 was not available, and consequently, the American Joint Committee on Cancer (AJCC) staging system was not adopted (14).

Based on ^18^F-FDG PET/CT alone, the disease extent was classified as follows: 1) tumor without nodal or distant metastasis; 2) tumor with nodal metastasis and no distant metastasis; and 3) tumor with nodal and distant metastases. To determine the actual clinical approach based on surgical resectability, the overall disease stage was re-categorized into curative (localized tumor + resectable nodal metastasis) and palliative (tumor + unresectable distant nodal metastasis ± distant metastasis), based on contrast CT findings and PET/CT reports.

### Survival follow-up

The overall survival was used as the primary endpoint to evaluate the prognostic significance. The overall survival was measured from the date of diagnosis of esophageal carcinoma to the date of patient’s death by any cause. The surviving patients were followed-up for at least 12 months. Among 40 patients, nine cases missed the survival data.

### Statistical analysis

SPSS version 19.0 was used for the statistical analysis. The mean SUV_max_ was analyzed, using unpaired t-test and ANOVA test. Survival after follow-up was analyzed, using the Kaplan–Meier product-limit method. P-value less than 0.05 was considered statistically significant.

## Results

### Baseline characteristics

Distribution of the characteristics of patients and tumors is summarized in Table 1. The median age of the subjects was 61 years (range: 42-78 years), and the male-to-female ratio was 24/16. Contrary to the reports in the United States, squamous cell carcinoma was the predominant histological subtype in the present study (15).

**Table 1:** Distribution of the characteristics of patients and tumors

Parameters	Characteristics	N	%
Sex	Male	24	60.0

	Female	16	40.0

Histology	Adenocarcinoma	15	37.5

	Squamous cell carcinoma	19	47.5

	Not available	6	15.0

Tumor location	Upper third	3	7.5

	Middle third	9	22.5

	Lower third	28 (16 EGJ*)	70.0

EGJ= Esophagogastric junction

In the AJCC staging manual (seventh edition), the previously classified esophagogastric junction (EGJ) tumor was re-categorized as lower third esophageal tumor. By this re-classification, the lower esophagus became the primary site of esophageal tumors (14).

### Correlation between SUV_max_ and tumor characteristics

The SUV_max_ of ^18^F-FDG in the primary lesion was high with the mean value of 12.6+7.1 (Table 2). A higher SUV_max_ was demonstrated in the proximal esophagus, compared to middle and lower regions (P=0.04). Expectedly, the squamous cell type predominantly exhibited higher SUV_max_, compared to adenocarcinoma cell type (P=0.02).

**Table 2:** Correlation between maximum standardized uptake value (SUV_max_), tumor characteristics, and tumor spread

Factors	Mean SUV_max_ (95% CI)	
**Tumor location**		
Upper (n=3)	21.3 (14.2, 28.3)	P=0.04
Middle (n=9)	13.6 (8.4, 18.7)	
Lower (n=28)	11.1 (8.7, 14.2)	

**Histology**		
Adenocarcinoma	9.7 (6.4, 13.0)	P=0.02
Squamous cell cancer	15.1 (12.0, 18.3)	

T involvement: tumor without nodal or distant metastasis (n=18)	10.6 (7.6, 13.7)	P=0.11
T + N involvement: tumor with nodal metastasis but no distant metastasis (n=7)	11.4 (5.4, 17.3)	
T + N + M involvement: tumor with nodal and distant metastases (n=15)	15.7 (11.3, 20.0)	

Localized tumor + resectable nodal metastasis (n=21)	10.4 (7.7, 13.1)	P=0.03
Tumor + unresectable distant nodal metastasis ± distant metastasis (n=19)	15.1 (11.4, 18.9)	

T = tumor, N= nodal, M=distant metastasis

With regard to the classification of disease extension, the mean SUV_max_ progressively increased from localized tumors to nodal metastases and subsequently to distant metastases, although this finding was not statistically significant. However, when the tumor stage was re-classified on the basis of surgical resectability, the unresectable group had a significantly higher SUV_max_, compared to the resectable group.

### Management impact of pre-therapeutic ^18^F-FDG PET/CT

Among 40 patients, ^18^F-FDG PET/CT led to a change in disease stage in 26 patients, with upstaging and downstaging reported in 10 and 16 cases, respectively (Table 3). Also, among 24 patients with palliative care as their initial treatment strategy, 10 cases were re-classified in the curative group on the basis of ^18^F-FDG PET/CT scan. On the other hand, management modification from curative to palliative was observed in 7 out of 16 patients (Table 3).

**Table 3:** Impact of ^18^F-FDG PET/CT on modifications in disease stage and therapy

Findings	N=40	Percentage
PET impact on disease stage	26	65.0%
Upstaging	10	25.0%
Downstaging	16	40.0%

PET impact on disease management	17/40	42.5%
From palliative to curative	10/24	41.7%
From curative to palliative	7/16	43.8%

**Figure 1:**
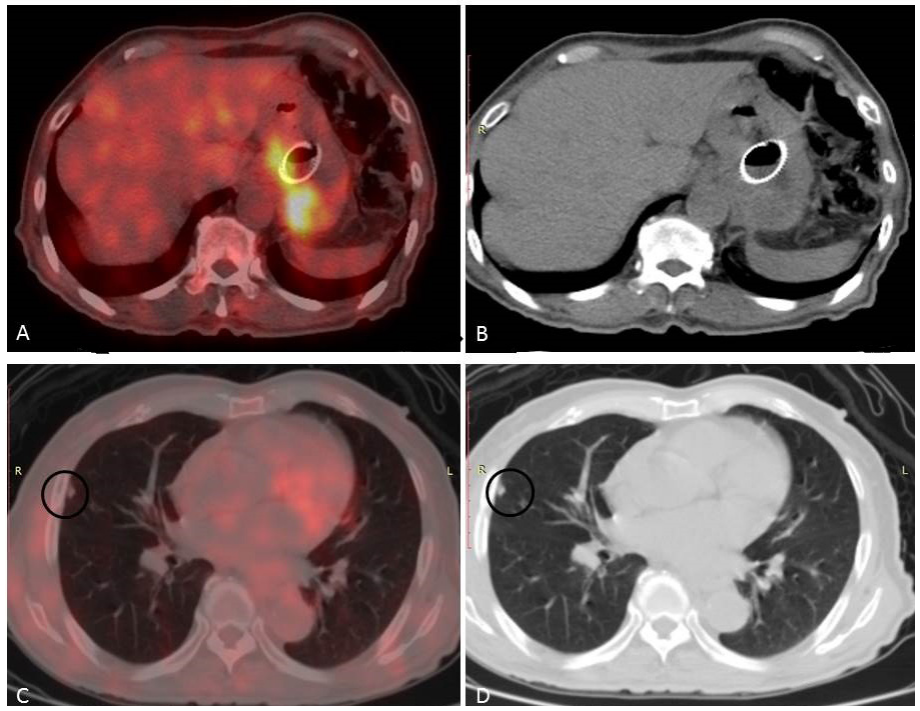
A 73-year-old lady with biopsy-proven gastroesophageal adenocarcinoma (moderately differentiated histological type) underwent ^18^F-FDG PET/CT to rule out solitary pulmonary metastasis. The PET/CT fusion image showed FDG hypermetabolism in the gastroesophageal junction with stent in-situ (a, b). However, no FDG hypermetabolism was demonstrated in the right upper lobe pulmonary nodule (c, d). The management strategy was shifted from palliative to curative. Esophagectomy was performed after radiotherapy on the primary lesion. The patient survived during the 12-month follow-up

**Figure 2:**
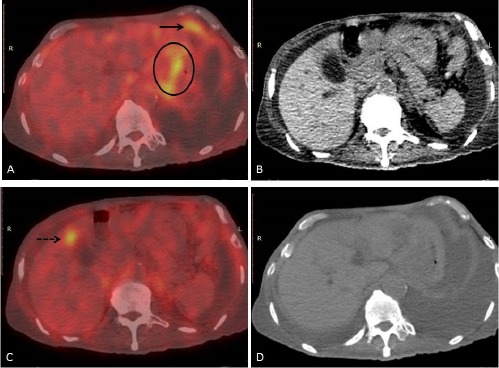
A 67-year-old man with biopsy-proven gastroesophageal adenocarcinoma underwent ^18^F-FDG PET/CT for further assessment. Initial staging CT scan showed a localized primary lesion at the gastroesophageal junction (not shown). However, subsequent PET/CT images showed FDG hypermetabolism at the gastroesophageal junction (circle), peritoneum (arrow), and liver (dotted arrow) (a-d). The management strategy was shifted from curative to palliative. No surgery was performed, and he passed away 3 months after PET/CT

Among 19 patients in the curative group, only eight cases underwent surgical resection with or without neoadjuvant chemoradiation, two patients opted for only chemoradiation, two patients refused treatment, and seven cases missed the follow-ups. On the other hand, in the palliative group, 12 out of 21 patients received chemoradiation therapy, seven patients refused treatment, and two patients missed the follow-ups. In 5 out of 24 patients, the management strategy was modified from palliative to curative, while in 5 out of 16 patients, the strategy changed from curative to palliative. Figures 1 and 2 shows two examples of treatment change.

**Figure 3:**
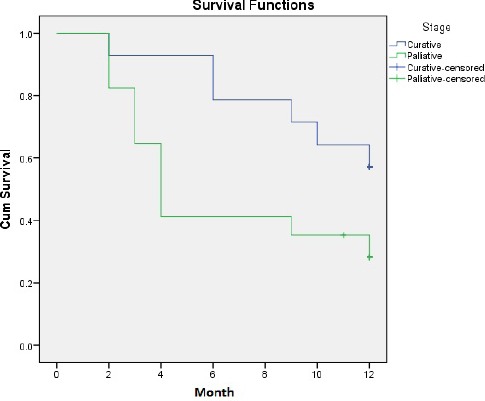
Survival of patients in curative and palliative groups based on ^18^F-FDG PET/CT alone

Based on ^18^F-FDG PET/CT alone, the median survival of patients in the palliative group was 4.0 (95% CI: 3.0-5.0) months, whereas the median survival of patients in the curative group has not been reached in the 12-month follow-up (Figure 3). The treatment strategy on the basis of ^18^F-FDG PET/CT alone was significantly associated with survival outcomes at nine months (P=0.03) and marginally significant at twelve months (P=0.05).

### Relationship between SUV_max_ and survival outcomes

In 31 patients with available survival data, the median SUV_max_ of the primary tumor was 10.1+4.9 (Table 4). Therefore, SUV_max_ of 10 was selected as the cut-off point while analyzing the survival outcomes in comparison with treatment factors and SUV_max_. Overall, the link between survival and SUV_max_ in surgically treated, chemoradiation, and non-treatment groups was not statistically significant. Moreover, it is interesting to note that in the chemoradiation group, a higher median survival was observed in patients with SUV_max_ above 10.

**Table 4:** Correlation between SUV_max_ and 12-month survival based on the treatment strategy

Treatment strategy	Median survival (month) (95% CI)	
Surgical resection		
SUV_max_ ≤10 (n=3)	NR	P=0.36
SUV_max_ > 10 (n=5)	10.0 (1.4, 18.6)	

Chemoradiotherapy		
SUV_max_ ≤ 10 (n=8)	4.0 (NA, NA)	P=0.90
SUV_max_ > 10 (n=6)	12.0 (NA, NA)	

No treatment
SUV_max_ ≤ 10 (n=4)	6.0 (0.1,11.9)	P=0.47
SUV_max_ > 10 (n=5)	4.0 (2.2, 5.8)	

NR = not reached, NA = not available

## Discussion

Over the past few years, ^18^F-FDG PET/CT has shown increasing efficacy in pre-operative staging of esophageal carcinoma, particularly in the detection of remote nodal and systemic metastatic diseases (16, 18). ^18^F-FDG PET/CT has an accuracy of 83.7% in detecting nodal metastases, while CT scan alone exhibits an accuracy of 76.6% (6). Furthermore, the accuracy of ^18^F-FDG PET/CT scan in detecting distant metastases supersedes CT scan (96.43% vs. 78.57%) (7). This level of accuracy is crucial, as 20-30% of patients with esophageal carcinoma with demonstrable metastatic disease at the time of initial diagnosis are precluded from high-risk curative surgeries (8).

In the present study, non-contrasted ^18^F-FDG PET/CT was used as an additional modality following conventional contrasted CT scan and EUS to further stage the disease, based on lymph node involvement (N) and distant metastasis (M) criteria. As a result, changes in disease stage were observed in 65.0% of patients with upstaging and downstaging in 25.0% and 40.0% of cases, respectively.

In addition, in the present study, the impact of ^18^F-FDG PET/CT scan on therapeutic changes was tremendous. In total, 41% of patients with the initial palliative strategy were re-classified in the curative group, whereas an opposite trend was reported in 31.3% of patients. These findings have clear implications for treatment decision-making, as nearly one-third of the patients were re-classified as either candidates who will benefit from curative surgery or prevented from unnecessary high-risk surgery.

In the present study, the high percentage of ^18^F-FDG PET/CT-induced changes in the management strategy is comparable with previous studies (10, 19-22). However, such results tend to overlook the fact that 27.5% of patients in this study were referred for confirmatory ^18^F-FDG PET/CT due to initial equivocal CT findings (e.g., solitary subcentimeter pulmonary nodule and small hypodense hepatic lesion).

As the frequency of patient referrals based on indeterminate CT findings increases, the likelihood of stage change after ^18^F-FDG PET/CT may be increased. Moreover, in this study, patients with T1 or T2 cancer were excluded from ^18^F-FDG PET/CT evaluation, as previous studies have revealed the insignificant diagnostic yield of PET in the detection of unsuspected metastatic disease at early stages (23). In summary, such selective referrals are likely to contribute to sampling bias. Nonetheless, despite such potential bias, the change in management strategy is inevitable, based on correct staging in the majority of patients.

Although the significant role of ^18^F-FDG PET/CT in therapeutic changes does not necessarily translate to survival benefit, several studies have shown the efficacy of this modality in providing prognostic information in esophageal carcinoma (22, 24). This finding was also highlighted in the current study, where PET-CT-guided treatment plan was significantly associated with 12-month survival outcomes.

The difference in survival outcomes between curative and palliative groups was obvious at nine months, based on ^18^F-FDG PET/CT alone. However, despite the fact that the median survival in the curative group has not been reached at 12-month follow-up, the survival outcomes in this period between the two groups were marginally different due to the convergence of both curves after nine months (P=0.05). This convergence might be related to the improved outcomes of patients who subsequently responded to the definitive chemoradiation regimens (25); however, longer duration of follow-ups is needed to confirm this finding.

Various studies with controversial results have been published on the relationship between SUV_max_ and survival outcomes in esophageal carcinoma (26). The median SUV_max_ values in various studies range from 0.26 to 17.2, as summarized by Taan et al. (26). In the current study, the mean value of SUV_max_ (12.6) was higher than the majority of conducted studies.

Also, the present findings were in agreement with the results reported by Taan et al., indicating the significantly higher value of SUV_max_ in squamous cell carcinoma subtype, which was predominantly located in the upper esophageal region (26). However, these results remain unexplained and assessment of the possible link between the biological factors of two cellular subtypes and glucose metabolism is an interesting subject for future research.

Furthermore, in the present study, similar to the findings reported by Taan et al., SUV_max_ was significantly elevated in the primary tumor as the disease burden (disease stage) increased (26). This finding was consistent with the notion that a large tumor burden is probably associated with a high tumoral proliferative rate and aggressiveness, which are in turn closely related to high glucose metabolism (27).

A meta-analysis by Pan et al. reported a hazard ratio of 1.86 by evaluating the prognostic value of SUV for the overall survival of patients with esophageal carcinoma (11). Interestingly, despite the close relationship between SUV_max_ and disease stage, the findings of this study showed a poor association between survival outcomes and SUV_max_ among surgically treated, chemoradiation, and non-treatment groups. Although these results were contrary to the findings reported by Pan et al., they were in line with other studies, showing that pre-treatment SUV_max_ is of limited use in prognostic stratification (28-31).

The main reason for the poor correlation between pre-treatment SUV_max_ and disease prognosis, as suggested by Taan et al., is the overriding effect of stage-based prognostic factor, which directly influences the therapeutic approach (26). Therefore, pre-treatment SUV_max_ does not provide relevant information, influencing the actual clinical decision-making. Other parameters such as functional tumoral length, functional tumor volume, and total lesion glycolysis may be relevant prognosticators for survival outcomes (28-32), which may be of interest in future studies.

The major limitations of the present study included the small sample size hindering multivariate analysis, significant missing data due to the retrospective design of the study, and disease misclassification bias (due to intrinsic limitation of PET/CT instrumentation, as well as ^18^FDG tracer). The use of contrasted agents during CT acquisition on ^18^F-FDG PET/CT could be useful in making direct comparisons with the initial contrasted CT findings too.

## Conclusion

^18^F-FDG PET/CT had a significant impact on stage stratification and subsequently, determination of the stage-stratified treatment approach in patients with esophageal carcinoma. Such stage-guided treatment strategies could improve the overall survival outcomes. However, pre-treatment SUV_max_ failed to be of use in the prognostic assessment and survival outcomes.

## Conflicts of interest

The authors declare none conflicts of interest.
